# SlDELLA interacts with SlPIF4 to regulate arbuscular mycorrhizal symbiosis and phosphate uptake in tomato

**DOI:** 10.1093/hr/uhae195

**Published:** 2024-07-21

**Authors:** Lan Li, Shibei Ge, Liqun He, Ruicheng Liu, Yuhong Mei, Xiaojian Xia, Jingquan Yu, Yanhong Zhou

**Affiliations:** Department of Horticulture, Zijingang Campus, Zhejiang University, 866 Yuhangtang Road, Hangzhou 310058, China; Department of Horticulture, Zijingang Campus, Zhejiang University, 866 Yuhangtang Road, Hangzhou 310058, China; Tea Research Institute, Chinese Academy of Agricultural Science, Hangzhou 310008, China; Department of Horticulture, Zijingang Campus, Zhejiang University, 866 Yuhangtang Road, Hangzhou 310058, China; Department of Horticulture, Zijingang Campus, Zhejiang University, 866 Yuhangtang Road, Hangzhou 310058, China; Hainan Institute, Zhejiang University, Sanya 572025, China; Department of Horticulture, Zijingang Campus, Zhejiang University, 866 Yuhangtang Road, Hangzhou 310058, China; Department of Horticulture, Zijingang Campus, Zhejiang University, 866 Yuhangtang Road, Hangzhou 310058, China; Key Laboratory of Horticultural Plant Growth and Development, Ministry of Agriculture and Rural Affairs of China, Hangzhou 310058, China; Department of Horticulture, Zijingang Campus, Zhejiang University, 866 Yuhangtang Road, Hangzhou 310058, China; Key Laboratory of Horticultural Plant Growth and Development, Ministry of Agriculture and Rural Affairs of China, Hangzhou 310058, China; Department of Horticulture, Zijingang Campus, Zhejiang University, 866 Yuhangtang Road, Hangzhou 310058, China; Hainan Institute, Zhejiang University, Sanya 572025, China; Key Laboratory of Horticultural Plant Growth and Development, Ministry of Agriculture and Rural Affairs of China, Hangzhou 310058, China

## Abstract

Arbuscular mycorrhizal symbiosis (AMS), a complex and delicate process, is precisely regulated by a multitude of transcription factors. PHYTOCHROME-INTERACTING FACTORS (PIFs) are critical in plant growth and stress responses. However, the involvement of PIFs in AMS and the molecular mechanisms underlying their regulator functions have not been well elucidated. Here, we show that SlPIF4 negatively regulates the arbuscular mycorrhizal fungi (AMF) colonization and AMS-induced phosphate uptake in tomato. Protein–protein interaction studies suggest that SlDELLA interacts with SlPIF4, reducing its protein stability and inhibiting its transcriptional activity towards downstream target genes. This interaction promotes the accumulation of strigolactones (SLs), facilitating AMS development and phosphate uptake. As a transcription factor, SlPIF4 directly transcriptionally regulates genes involved in SLs biosynthesis, including *SlCCD7*, *SlCDD8,* and *SlMAX1*, as well as the AMS-specific phosphate transporter genes *PT4* and *PT5*. Collectively, our findings uncover a molecular mechanism by which the SlDELLA-SlPIF4 module regulates AMS and phosphate uptake in tomato. We clarify a molecular basis for how SlPIF4 interacts with SLs to regulate the AMS and propose a potential strategy to improve phosphate utilization efficiency by targeting the AMS-specific phosphate transporter genes *PTs*.

## Introduction

Phosphorus (P), one of the essential macronutrients, is of indispensable importance in promoting plant growth and development, as well as improving the yield and quality of crops. Although phosphate (Pi) is abundant in agricultural soils, most of it cannot be directly absorbed by plants due to its low mobility and availability [1, 2]. When faced with an unfavorable environment, plants can establish various mutually beneficial relationships with microorganisms, especially with arbuscular mycorrhizal fungi (AMF) to form symbionts, called arbuscular mycorrhizal symbiosis (AMS) [3, 4]. Through AMS, the host plant acquires available Pi, water, and other essential nutrients, thereby improving its stress resistance [5–8]. In turn, AMF achieves growth and reproduction by obtaining carbon materials including fatty acids and sugars, provided by the host plant [9, 10]. Therefore, it is important and highly meaningful to clarify the regulatory mechanism of AMS to improve the utilization efficiency of phosphate and achieve the sustainable development of agriculture.

AMS is a complex and delicate process, including the signaling dialog at the pre-contact stage, the penetration process and arbuscule formation, as well as the arbuscule development and degeneration, which are precisely regulated by phytohormones, especially strigolactones (SLs) [11, 12]. Root-exudated SLs stimulate the germination, hyphae branching, and elongation of AMF spores existing in soil, thus promoting AMS [13–16]. Previous studies also showed that light signaling influenced the colonization of AMF. Compared with low Red/Far-Red light, high Red/Far-Red light significantly promoted the colonization of AMF in tomato and *Lotus japonicus* [17]. While we previously revealed that the main red-light photoreceptor phyB promotes the accumulation of LONG HYPOCOTYL 5 (HY5, a light signal transcriptional factor) protein in tomato roots, which further controls the AMS by systemically regulating SLs biosynthesis [18], the mechanism underlying light-regulated AMS has not yet been fully revealed. It will be interesting to explore more molecular players that integrate light and hormone signals to regulate AMS.

PIFs (PHYTOCHROME-INTERACTING FACTORS), light-regulated bHLH transcription factors, play an important role in plant growth and development [19–21]. There are eight PIFs in *Arabidopsis thaliana* and tomato, respectively [22–24]. All PIFs consist of an APB (active phyB-binding) motif, and a bHLH functional domain including the basic DNA-binding domain and the helix–loop–helix (HLH) domain [25]. PIF4 is the regulatory center of various environmental signals and endogenous hormone signals involved in regulating plant growth and development as well as stress tolerance. Previous studies indicated that PIF4 not only directly induced or reduced the transcript levels of downstream genes, but also interacted with many proteins to regulate downstream response [26, 27]. Until now, it has been demonstrated that 25 proteins physically interact with PIF4, including photoreceptors, hormone signaling components, and so on [27–30]. However, the study of PIF4 in regulating AMS remains rudimentary.

DELLA, a plant-specific GRAS protein, not only mediates Gibberellins (GAs) signaling but also interacts with diverse transcription factors in many signaling pathways to interfere with or modulate their functions [31, 32]. Emerging evidence suggests that DELLA protein, as a central hub, is of great importance in controlling AMS development. In pea, DELLA-deficient *la cry-s* mutants show reduced arbuscular mycorrhizal colonization [33]. Similarly, mycorrhizal colonization is impaired obviously in *DELLA* mutants in *Medicago truncatula* and *Oryza sativa* [34, 35]. Additionally, DELLA proteins form a complex with certain transcription factors to influence the AMS. DELLA interacts with CYCLOPS, a component of the common symbiosis signaling pathway, and together with CCaMK (calcium and calmodulin-dependent kinase), they form a complex that activates *RAM1* expression, thereby regulating arbuscular development [36]. DELLA proteins were also revealed to interact with MYB1 to regulate arbuscule degeneration [37]. Previous studies demonstrated that DELLA could degrade PIFs protein through the 26S proteasome or sequestrate them from target genes to regulate PIFs transcriptional activity and protein stability [38, 39]. Although DELLA is a key component in the transcription network to regulate AMS, the relationship between DELLA and PIF4 in AMS development remains unknown and needs further study.

In this study, we show that SlPIF4 negatively regulates the AMS and AMS-induced Pi uptake in tomato, which is contrary to the function of SlDELLA. Moreover, our data demonstrates that SlDELLA physically interacts with SlPIF4 to induce the expression of SlPIF4 targeted genes, including SLs biosynthesis genes and AMS-specific phosphate transporter genes *PTs*, by reducing the protein stability and attenuating the transcriptional activity of SlPIF4. Together, our findings unravel a novel mechanism regarding the SlDELLA-SlPIF4 module-mediated AMS and Pi uptake via an SLs-dependent pathway in tomato plants.

## Results

### *SlPIF4* negatively regulates the arbuscular mycorrhizal symbiosis in tomato

**Figure 1:**
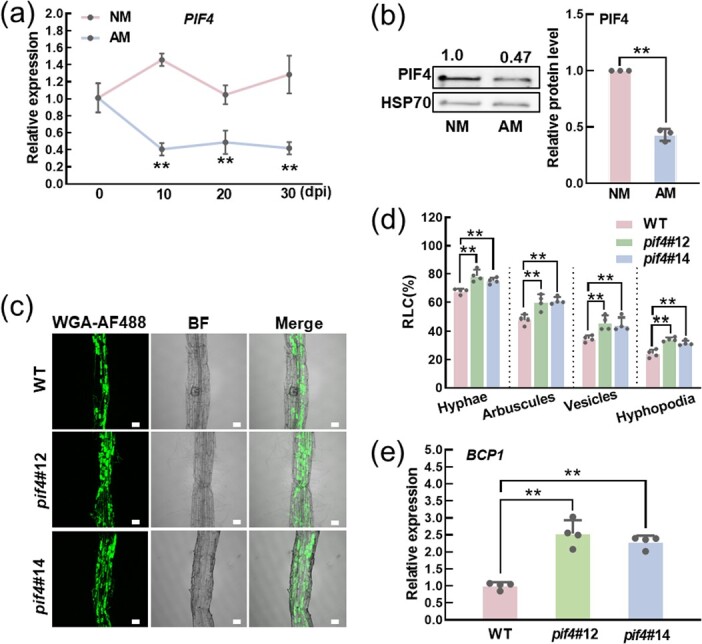
*SlPIF4* negatively regulates the AMF colonization in tomato. **(a)** Temporal expression pattern of *SlPIF4* in roots of WT plants. Dpi: days-post-inoculation. Data are presented as the means of three biological replicates (±SD). Asterisks indicate a statistically significant difference from the control in the means (**P < 0.01; Student’s *t* test). **(b)** Immunoblots showing the SlPIF4 protein level in roots of WT plants at 10 dpi. The same membrane was split into two sheets and incubated with anti-PIF4 and anti-HSP70 antibodies, respectively. Representative pictures are shown on the left. The relative protein levels are shown on the right of the blots, and the relative protein levels of WT in NM roots were set to 1.00. Data are presented as the means of three biological replicates (±SD). Asterisks indicate a statistically significant difference from the control in the means (**P < 0.01; Student’s *t* test). **(c)** Representative images of WGA-AF488-stained roots of WT and *pif4* mutant plants at 20 dpi. BF, bright-field image. Merge, merged WGA-AF488 and BF image. Scale bar = 100 μm. **(d)** The root length colonization of hyphae, arbuscules, vesicles, and hyphopodia in the roots of WT and *pif4* mutant plants at 20 dpi. Data are presented as the means of four biological replicates (±SD). Each replication contains 80 root segments of two plants. Asterisks indicate a statistically significant difference from the control in the means (^**^P < 0.01; Student’s *t* test). **(e)** Transcript of AMS-marker gene *BCP1* in roots of WT and *pif4* mutant plants at 20 dpi. Data are presented as the means of four biological replicates (±SD). Asterisks indicate a statistically significant difference from the control in the means (^**^P < 0.01; Student’s *t* test). For (a–e), the plants were grown in a sterilized soil: quartz sand: vermiculite mixture (1:1:1) inoculating with (AM) or without (NM) *R. intraradices* under Pi deficient condition (without KH_2_PO_4_ but with the addition of 1 mM KCl).

Eight genes encoding proteins in tomato have been identified that are similar to PIFs in *A. thaliana* [24]. Here, we detected the transcript levels of *SlPIFs* in *Rhizophagus intraradices* inoculated (AM) and non-inoculated (NM) roots of wild-type (WT) plants that grow under phosphate deficiency at 10 days post-inoculation (dpi). The AMS-marker genes *BCP1 (BLUE COPPER-BINDING PROTEIN1)*, *PT4,* and *PT5 (PHOSPHATE TRANSPORTER 4 and 5)* were significantly induced in AM roots compared with WT NM roots (Fig. S1a, see online supplementary material). Importantly, the transcript levels of several *SlPIFs* declined in AM roots, whereas the expression level of *SlPIF4* was the most significant (Fig. S1b, see online supplementary material). As the inoculation period extended, the root length colonization (RLC) of hyphae, arbuscules, and vesicles was consistently increased (Fig. S2a, see online supplementary material). However, the transcript level of *SlPIF4* in AM roots decreased sharply at the initial stage, which was only 0.4 times of the initial level at 10 dpi, while the transcript level in NM roots showed minimal fluctuation. (Fig. 1a). At the same time, the abundance of SlPIF4 protein in WT AM roots was observably lower than that in NM roots at 10 dpi (Fig. 1b). All these results indicated that *SlPIF4* may participate in regulating the AMS in tomato.

To further explore the role of *SlPIF4* in mediating AMS, one *SlPIF4* overexpression line (*PIF4*#89) and two *SlPIF4* loss-of-function mutants (*pif4*#12 and *pif4*#14) using CRISPR/Cas9 were used in this study [24]. Compared with WT plants, the AM roots of two *pif4* mutants (*pif4*#12 and *pif4*#14*)* exhibited greater RLC and a higher number of mature arbuscule structures, whereas the *SlPIF4* overexpression plants (*PIF4*#89) showed less at 20 dpi (Fig. 1c–d;  S3a–b, see online supplementary material). Although both WT, *pif4* mutants, and *SlPIF4* overexpressing plants could form fully developed arbuscules, the arbuscule in *pif4* mutants was larger than those in WT plants, whereas the opposite was observed in *SlPIF4* overexpressing plants (Fig. S3d, see online supplementary material). The results were consistent with further observations made from WGA-AF488 stained images (Fig. S3e, see online supplementary material). In AM roots, the transcript level of AMS-marker gene *BCP1* in *pif4*#12 and *pif4*#14 was much higher than in WT plants, which was opposite in *PIF4*#89 plants (Fig. 1e; Fig.  S3c, see online supplementary material). These data demonstrated that *SlPIF4* was a negative regulator of the AMS in tomato plants.

### SlDELLA interacts with SlPIF4 *in vivo* and in *vitro* and positively regulates AMS in tomato

**Figure 2:**
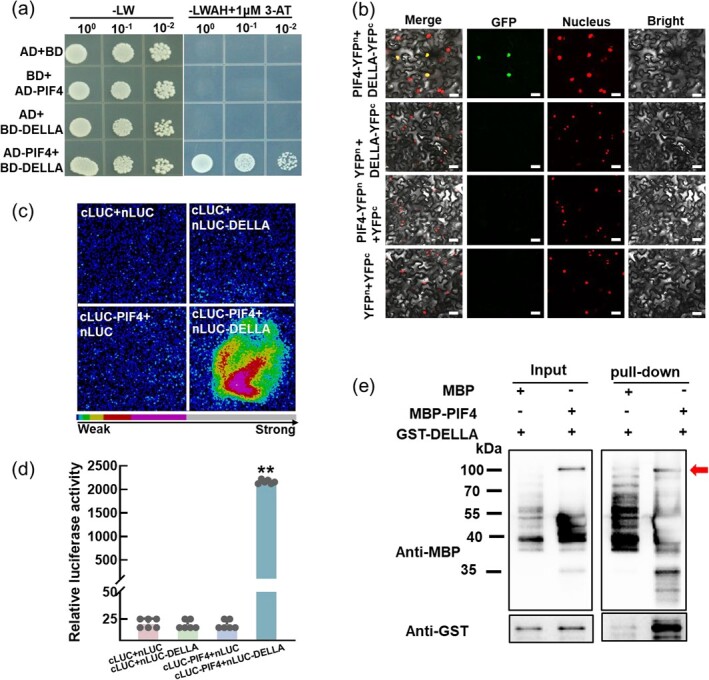
SlDELLA physically interacts with SlPIF4 *in vivo* and in *vitro*. **(a)** Yeast two-hybrid assay to confirm the interaction between SlDELLA and SlPIF4. Yeast cells were grown on SD/−Leu/−Trp (-LW) for 2 days or SD-Leu/−Trp/−Ade/-His (-LWAH) medium with 1 μM 3-AT for 5 days. **(b)** BiFC assay showing the interaction of SlDELLA with SlPIF4 in *Nicotiana benthamiana*. Full-length SlPIF4 and SlDELLA were fused to YFP^n^ or YFP^c^, respectively. Scale bar = 25 μm. Images were taken using a confocal microscope. H2B-mCherry was used as a nuclear marker. (**c**)–(**d**) Split luciferase complementation assay (SLCA) showing the interaction of SlPIF4 with SlDELLA. The cLUC-SlPIF4 and nLUC-SlDELLA constructs were co-transformed into *N. benthamiana* leaves, and the luminescence intensity was detected after 48 h. The luminescence intensity detected in *N. benthamiana* leaves co-expressed cLUC and nLUC as the control. Representative photographs are shown in **(c)**, and luciferase activity is shown in **(d)**. Data are presented as the means of six biological replicates (±SD). Asterisks indicate a statistically significant difference from the control in the means (^**^P < 0.01; Student’s *t* test). **(e)** Pull-down assay showing that MBP-tagged SlPIF4, but not MBP alone, could pull down GST-tagged SlDELLA in *vitro*. Recombinant MBP-SlPIF4 and GST-SlDELLA were detected with anti-MBP and anti-GST, respectively. The red arrow represents MBP-SlPIF4.

Considering DELLA-PIF modules integrate GA and light signals in *Arabidopsis*, we dissected the relationship between SlDELLA and SlPIF4 in tomato [38, 39]. Yeast two-hybrid (Y2H) analysis showed that only the yeast cells harboring SlPIF4-AD and SlDELLA-BD could grow on the medium (-LWAH) with 1 μM 3-AT, indicating that SlPIF4 could interact with SlDELLA (Fig. 2a). Further, as shown in the results of bimolecular fluorescence complementation (BiFC) assay, there were strong fluorescence signals when SlDELLA-YFP^c^ and SlPIF4-YFP^n^ co-expressed in *Nicotiana benthamiana* leaves. However, we could not detect fluorescence signals in other leaves co-expressing YFP^c^ and SlPIF4-YFP^n^, SlDELLA-YFP^c^ and YFP^n^, as well as YFP^n^ and YFP^c^ (Fig. 2b). Luciferase complementation assay (SLCA) also confirmed this interaction. Strong luciferase activity was detected only in co-expression combination with cLUC-SlPIF4 and nLUC-SlDELLA, reaching nearly 170 times the level observed in the control co-expressed cLUC and nLUC (Fig. 2c–d). Moreover, glutathione-S-transferase (GST) pull-down assay revealed that MBP-SlPIF4 fusion protein was capable of pulling down GST-SlDELLA fusion protein, rather than MBP protein (Fig. 2e).

Since DELLA proteins function as a central node to control the AM development [36, 40], we then detected the expression level and protein abundance of SlDELLA in NM and AM roots of WT plants at 10 dpi. Although there was no difference in the transcript level of *SlDELLA* between the NM roots and AM roots, the protein abundance was significantly increased in WT AM roots at 10 dpi in comparison with that in NM roots (Fig. S4a–c, see online supplementary material). We further used *procera* (*pro*) mutant, a putative DELLA mutant [41], and WT plants to test the involvement of SlDELLA in regulating AMS. The results indicated that there were fewer hyphae, arbuscules, and vesicles in *pro* roots in comparison with WT roots at 20 dpi (Fig. S5b, see online supplementary material). The results were compatible with the WGA-AF488 staining images (Fig. S5a, see online supplementary material). The transcript level of *BCP1* was increased in AM roots of WT plants, but significantly decreased in *pro* roots at 20 dpi (Fig. S5c, see online supplementary material). Collectively, these data demonstrated that SlDELLA physically interacts with SlPIF4 and positively regulates the AMF colonization in tomato.

### SlDELLA acts upstream of SlPIF4 to regulate arbuscular mycorrhizal symbiosis

**Figure 3:**
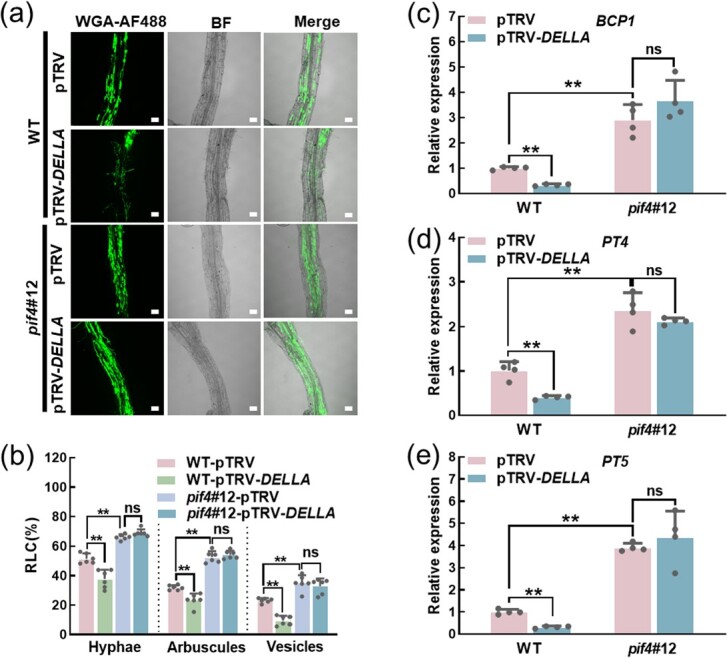
SlDELLA acts upstream of SlPIF4 to regulate the AMS in tomato. **(a)** Representative images of WGA-AF488-stained roots of WT-pTRV, WT-pTRV-*SlDELLA*, *pif4*#12-pTRV, and *pif4*#12-pTRV-*SlDELLA* plants at 20 dpi. BF, bright-field image. Merge, merged WGA-AF488 and BF image. Scale bar =100 μm. **(b)** The root length colonization of hyphae, arbuscules, and vesicles in the roots of WT-pTRV, WT-pTRV-*SlDELLA*, *pif4*#12- pTRV, and *pif4*#12-pTRV-*SlDELLA* plants at 20 dpi*.* Data are presented as the means of six biological replicates (±SD). Each replication contains 80 root segments of two plants. Asterisks indicate a statistically significant difference from the control in the means (**P < 0.01; Student’s *t* test); ns: no significant difference. **(c)**–**(e)** Transcripts of AMS-marker genes *BCP1, PT4*, and *PT5* in roots of WT-pTRV, WT-pTRV-*SlDELLA*, *pif4*#12-pTRV, and *pif4*#12-pTRV-*SlDELLA* plants at 20 dpi. Data are presented as the means of four biological replicates (±SD). Asterisks indicate a statistically significant difference from the control in the means (^**^P < 0.01; Student’s *t* test); ns: no significant difference. For **(a)**–**(e)**, the plants inoculated with *R. intraradices* were grown in a sterilized soil: quartz sand: vermiculite mixture (1:1:1) under Pi deficient condition (without KH_2_PO_4_ but with the addition of 1 mM KCl).

To clarify the genetic relationship of SlDELLA and SlPIF4, we silenced *SlDELLA* in WT and *pif4#12* plants using virus-induced gene silencing (VIGS) technology. The plants with 70–80% silencing efficiency were used in the study (Fig. S6a, see online supplementary material). The deletion of *SlPIF4* and reduction of *SlDELLA* significantly increased and decreased the RLC of hyphae, arbuscules, and vesicles compared with the WT-pTRV plants at 20 dpi, respectively (Fig. 3b), which also be clearly reflected in the images of WGA-AF488 staining (Fig. 3a). These results indicated that SlPIF4 and SlDELLA played opposite roles in regulating the mycorrhizal colonization. However, silencing *SlDELLA* in the *pif4* mutant (*pif4*#12) plants did not result in a decrease in mycorrhizal colonization rate compared with *pif4*#12 plants. The mycorrhizal colonization level of the *pif4*#12-pTRV-*SlDELLA* plants was almost the same as that of *pif4*#12 plants (Fig. 3a–b). Similarly, the transcript levels of AMS-marker genes (*BCP1*, *PT4*, and *PT5*) were lower in pTRV-*SlDELLA* plants and higher in *pif4*#12-pTRV than in WT-pTRV plants. Importantly, when silencing *SlDELLA* in *pif4*#12-pTRV plants, there was no difference in the transcript levels of these tested AMS-marker genes compared with the *pif4*#12-pTRV plants (Fig. 3c–e). These results indicated that SlDELLA acts upstream of SlPIF4 to regulate the AMS in tomato.

### *SlPIF4*-mediated AMS is partly dependent on SLs biosynthesis, accumulation, and exudation in tomato roots.

**Figure 4:**
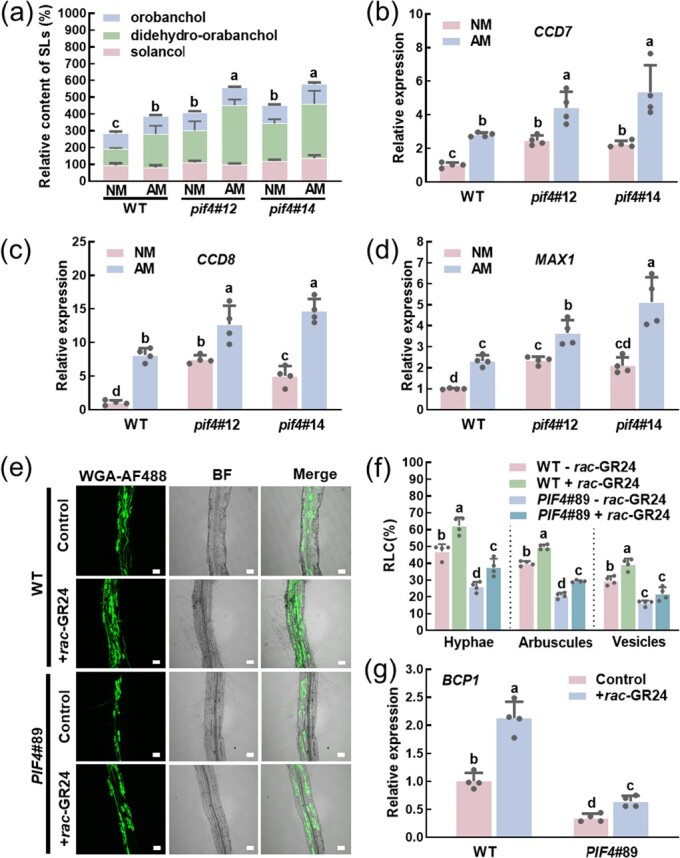
*SlPIF4*-mediated AMS is partly dependent on SLs biosynthesis, accumulation and exudation in tomato. **(a)** Relative contents of SLs from WT and *pif4* mutant plants roots at 10 dpi. Data are presented as the means of three biological replicates (±SD). Different letters indicate significant differences by One-way ANOVA followed by post *hoc* Tukey test (*P* < 0.05). **(b)**–**(d)** The expression of *SlCCD7*, *SlCCD8*, and *SlMAX1* in roots of WT and *pif4* mutant plants at 10 dpi. Data are presented as the means of four biological replicates (±SD). Different letters indicate significant differences by One-way ANOVA followed by post *hoc* Tukey test (*P* < 0.05). **(e)** Representative images of WGA-AF488-stained roots of in WT and *SlPIF4*-overexpressing (*PIF4*#89) plants applied with or without 1 μM *rac*-GR24 at 20 dpi. BF, bright-field image. Merge, merged WGA-AF488 and BF image. Scale bar =100 μm. **(f)** The root length colonization of hyphae, arbuscules, and vesicles in the roots of WT and *SlPIF4*-overexpressing (*PIF4*#89) plants applied with or without 1 μM *rac*-GR24 at 20 dpi*.* Data are presented as the means of four biological replicates (±SD). Each replication contains 80 root segments of two plants. Different letters indicate significant differences by one-way ANOVA followed by post *hoc* Tukey test (*P* < 0.05). **(g)** Transcripts of AMS-marker gene *BCP1* in roots of WT and *SlPIF4*-overexpressing (*PIF4*#89) plants applied with or without 1 μM *rac*-GR24 at 20 dpi. Data are presented as the means of four biological replicates (±SD). Different letters indicate significant differences by One-way ANOVA followed by post *hoc* Tukey test (*P* < 0.05). For **(a)**–**(d)**, the plants inoculated with (AM) or without (NM) *Rhizophagus intraradices* were grown in a sterilized soil: quartz sand: vermiculite mixture (1:1:1) under Pi deficient condition (without KH_2_PO_4_ but with the addition of 1 mM KCl). For **(e)**–**(g)**, the WT and *SlPIF4*-overexpressing (*PIF4*#89) plants inoculated with *R. intraradices* were treated with ddH_2_O (Control) or + *rac*-GR24 (1 μM) three times a week.

Under Pi deficiency, the host plants will synthesize and secrete SLs into the soil [42, 43]. Secreted SLs stimulate the AMF spore germination, hyphal branching, chitin oligomer production, and soon, thus facilitating symbiosis [16, 44, 45]. Based on this, we used two *SlPIF4* mutants and WT plants to explore the relationship between *SlPIF4* and SLs. Under sufficient Pi conditions, the deletion of *SlPIF4* apparently increased the relative content of root-exudated SLs (Fig. S7a, see online supplementary material). Importantly, the SLs relative content and the transcript levels of *SlCCD7*, *SlCCD8*, and *SlMAX1* in AM roots of *pif4#*12 and *pif4#*14 plants were higher than WT plants at 10 dpi (Fig. 4a–d). In addition, Pi starvation also induced the biosynthesis and root exudation of SLs (Fig. S7a–d, see online supplementary material). On the contrary, the *PIF4*#89 plants displayed a decrease in SLs content and the transcript levels of *SlCCD7*, *SlCCD8*, and *SlMAX1* both in NM and AM roots, and the downtrend was more significant in AM roots (Fig. S8a–d).

To demonstrate that SLs were regulated by *SlPIF4* in AMS development, the synthetic SL *rac*-GR24 and *SlPIF4* overexpression plants were used in the study. At 20 dpi, *rac*-GR24 application significantly increased the colonization of AMF in WT plants showing more hyphae, arbuscules, and vesicles (Fig. 4e–f). Similarly, the transcript levels of *BCP1*, *PT4*, and *PT5* were also increased by *rac*-GR24 (Fig. 4g; Fig.  S8e and f, see online supplementary material). In comparison with the WT plants, the AMF colonization was decreased in *PIF4#89* plants. Significantly, the defects of AMF colonization and transcription of related genes in *PIF4#89* plants can restore partially by the application of *rac*-GR24 (Fig. 4e–g; Fig.  S8e and f, see online supplementary material). Taken together, *SlPIF4* negatively regulated the AMS in tomato by modulating the biosynthesis and secretion of SLs.

### SlPIF4 directly binds to and inhibits the promoters of strigolactones biosynthesis genes

**Figure 5:**
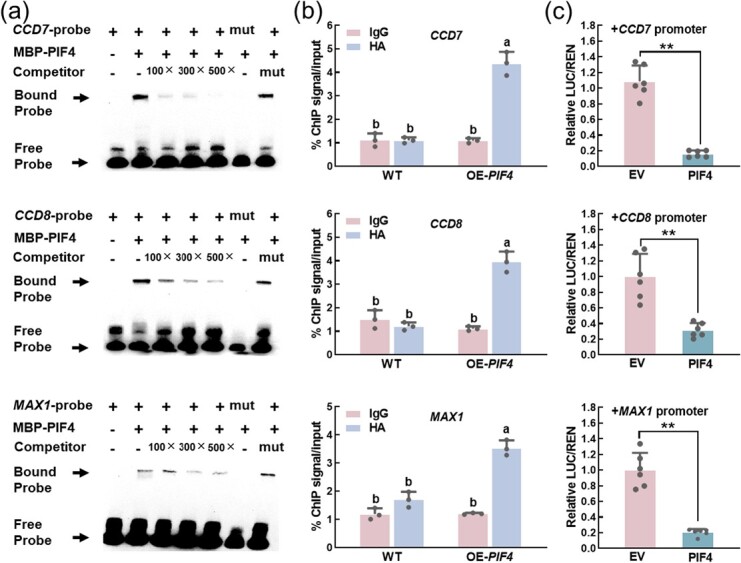
SlPIF4 directly binds to promoters of SLs biosynthesis genes and inhibits its transcription. **(a)** EMSA assay showing the binding of SlPIF4 to the promoters of *SlCCD7*, *SlCCD8*, and *SlMAX1*. Excess amounts (100×, 300×, 500×) of non-labeled or mutant oligo (mut) were set as the competitors. The biotin non-labeled oligo was used as a competitor, −: absence; +: presence. **(b)** ChIP-qPCR showing the binding of SlPIF4 to the *SlCCD7*, *SlCCD8*, and *SlMAX1* promoters containing the G/E-box motifs in *vivo*. HA, hemagglutinin; OE, overexpressing. Values are percentages of DNA fragments that coimmunoprecipitated with anti-HA antibodies or anti-IgG relative to the input DNA. Data are presented as the means of three biological replicates (±SD). Different letters indicate significant differences by one-way ANOVA followed by post *hoc* Tukey test (*P* < 0.05). **(c)** Dual-luciferase assay showing the inhibition of SlPIF4 to the *SlCCD7*, *SlCCD8* and *SlMAX1* promoters in *Nicotiana benthamiana*. EV: empty vector. The LUC/REN ratios of the empty vector (EV) plus promoter were set at ‘1’. Data are presented as the means of six biological replicates (±SD). Asterisks indicate a statistically significant difference from the control in the means (^**^P < 0.01; Student’s *t* test).

It has been suggested that PIF4 could bind to the G/E-box motifs (CANNTG) to modulate downstream gene expression [46]. There are two E-box motifs in the promoters of *SlCCD7*, *SlCCD8*, and *SlMAX1*, respectively, while there is only one G-box motif in *SlCCD8* and *SlMAX1* promoters, respectively (Fig. S9, see online supplementary material). Therefore, recombinant MBP-SlPIF4 protein and SLs biosynthesis genes-related probes were used in EMSA assay. MBP-SlPIF4 could bind to the *SlCCD7* and *SlCCD8* probes with E-box motif and the *SlMAX1* probe with G-box motif, respectively (Fig. 5a). However, the bound probes decreased gradually with the increase of competitors, and there were no bound probes when the probes lacked E/G-box motif (Fig. 5a). Then, in chromatin immunoprecipitation (ChIP) assay, AMF-inoculated *SlPIF4* overexpressing (*PIF4-OE*) and WT plants at 10 dpi were used to examine the binding ability of SlPIF4 *in vivo*. After being immunoprecipitated with anti-HA antibody, the promoter fragments of *SlCCD7*, *SlCCD8*, and *SlMAX1* in OE-*SlPIF4* lines were 4.4, 3.9, and 3.5 times more abundant than those in WT plants, respectively. On the contrary, there were no combinations of SlPIF4-HA and DNA fragments that could be immunoprecipitated after immunoprecipitation with IgG control antibody (Fig. 5b). Finally, dual-luciferase transient expression (LUC) assay demonstrated that SlPIF4 significantly inhibited the transcripts of *SlCCD7*, *SlCCD8*, and *SlMAX1* (Fig. 5c). Taken together, SlPIF4 could directly bind to the *SlCCD7*, *SlCCD8*, and *SlMAX1* promoters to suppress their transcription.

### SlPIF4 negatively regulates the AMS-induced phosphate uptake by inhibiting the transcription of AMS-specific *PTs*

**Figure 6:**
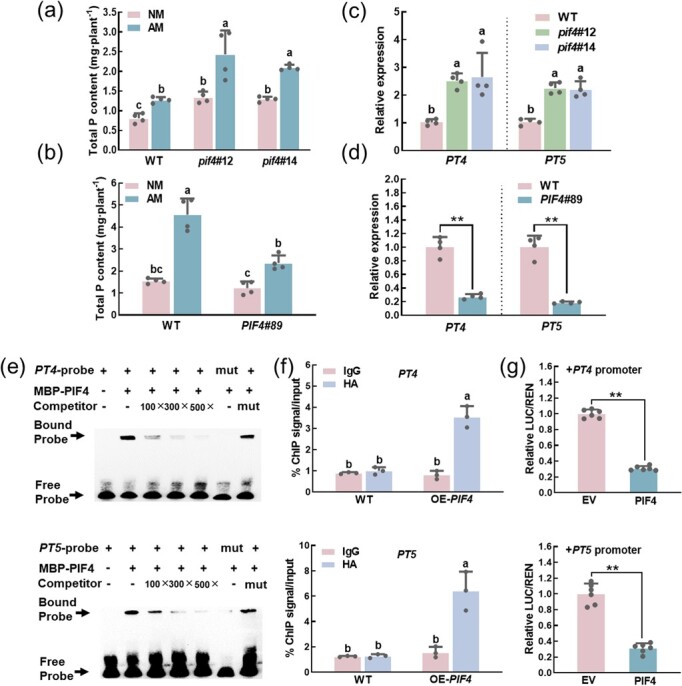
SlPIF4 negatively regulates the AMS-induced phosphate uptake by inhibiting the transcription of AMS-specific *PTs*. **(a)**–**(b)** Total P content of WT, *SlPIF4* overexpressing and *pif4* mutant plants at 30 dpi. Data are presented as the means of four biological replicates (±SD). Different letters indicate significant differences by one-way ANOVA followed by post *hoc* Tukey test (*P* < 0.05). **(c)**–**(d)** Transcripts of *PT4* and *PT5* in roots of WT, *SlPIF4* overexpressing (*PIF4*#89) and *pif4* mutant plants at 20 dpi*.* Data are presented as the means of four biological replicates (±SD). Different letters indicate significant differences by one-way ANOVA followed by post *hoc* Tukey test (*P* < 0.05). For **(a)**–**(d)**, the plants inoculated with (AM) or without (NM) *R. intraradices* were grown in a sterilized soil: quartz sand: vermiculite mixture (1:1:1) under Pi deficient condition (without KH_2_PO_4_ but with the addition of 1 mM KCl). **(e)** EMSA assay showing the binding of SlPIF4 to the promoters of AMS-specific *PTs*. Excess amounts (100×, 300×, 500×) of non-labeled or mutant oligo (mut) were set as the competitors. The biotin non-labeled oligo was used as a competitor, −: absence; +: presence. **(f)** ChIP-qPCR showing the binding of SlPIF4 to the AMS-specific *PTs* promoters containing the E-box motifs in *vivo*. HA, hemagglutinin; OE, overexpressing. Values are percentages of DNA fragments that coimmunoprecipitated with anti-HA antibodies or anti-IgG relative to the input DNA. Data are presented as the means of three biological replicates (±SD). Different letters indicate significant differences by one-way ANOVA followed by post *hoc* Tukey test (*P* < 0.05). **(g)** Dual-luciferase assay showing the inhibition of SlPIF4 to the AMS-specific *PTs* promoters in *Nicotiana benthamiana*. EV: empty vector. The LUC/REN ratios of the empty vector (EV) plus promoter were set at ‘1’. Data are presented as the means of six biological replicates (±SD). Asterisks indicate a statistically significant difference from the control in the means (^**^P < 0.01; Student’s *t* test).

AMS is an adaptive strategy for plants to improve phosphate uptake under phosphate-deficient conditions. PT4 and PT5, the AMS-inducible phosphate transporters, were proposed to import phosphate on the symbiotic interface [10, 47]. Compared with WT NM plants, the content of phosphorus increased in AM plants at 30 dpi. Surprisingly, AMS-induced phosphorus accumulation was drastically promoted in *pif4*#12 and *pif4*#14 plants and restrained in *PIF4*#89 plants (Fig. 6a and b). Consistently, the transcripts of the AMS-specific phosphate transporter genes *PT4* and *PT5* were lower in the *PIF4*#89 plants, but higher in the roots of *pif4*#12 and *pif4*#14 plants than in WT plants (Fig. 6c and d). These results suggested that *SlPIF4* deletion could promote AMS-specific phosphate transporter genes expression, and thus the phosphate uptake in tomato.

To dissect whether SlPIF4 influence the transcript levels of AMS-specific *PTs* by binding to their promoters, we analysed the promoters of *PTs*, and found two and three E-box motifs were present in the promoters of *PT4* and *PT5*, respectively (Fig. S10, see online supplementary material). EMSA assay was performed to confirm that SlPIF4 could respectively bind to the E-box motif of *PT4* and *PT5*, but unable to bind to mutant probes (Fig. 6e). The bound probes decreased gradually with the increase of competitors (Fig. 6e). Furthermore, ChIP-qPCR analysis substantiated that SlPIF4 binds to the *PT4* and *PT5* promoters (Fig. 6f). We finally carried out a dual-luciferase assay to show that SlPIF4 could inhibit the transcription of *PT4* and *PT5* (Fig. 6g). Taken together, SlPIF4 could directly bind to the G/E box motifs of AMS-specific *PTs* promoters to reduce genes expression and suppress their transcription.

### SlDELLA improves the expression of SlPIF4 target genes by reducing the protein stability and attenuating the transcriptional activity of SlPIF4

**Figure 7:**
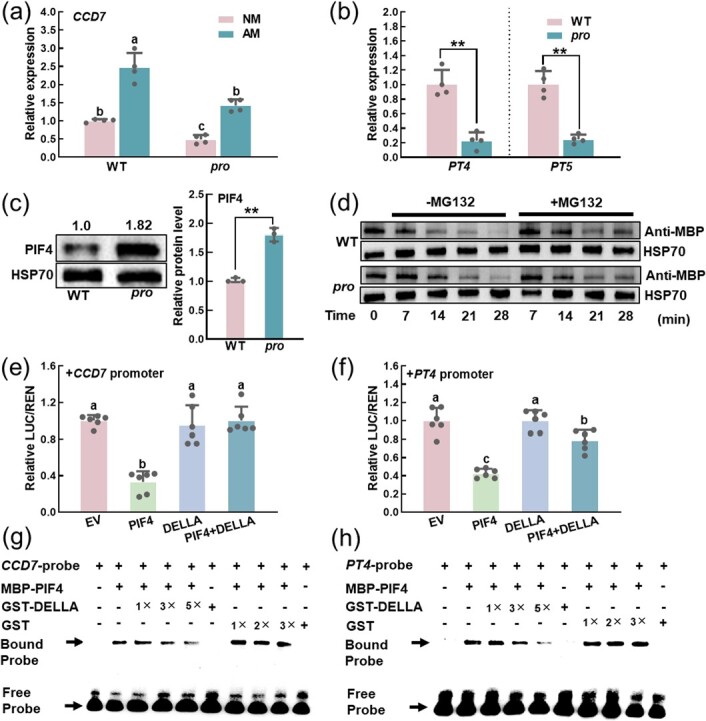
SlDELLA regulates the expression of SlPIF4 target genes by reducing the protein stability and attenuating the transcriptional activity of SlPIF4. **(a)** Transcript of *SlCCD7* in roots of WT and *pro* plants at 10 dpi*.* Data are presented as the means of four biological replicates (±SD). Different letters indicate significant differences by one-way ANOVA followed by post *hoc* Tukey test (*P* < 0.05). **(b)** Transcripts of AMS-specific *PTs* in roots of WT and *pro* plants at 20 dpi*.* Data are presented as the means of four biological replicates (±SD). Asterisks indicate a statistically significant difference from the control in the means (^**^P < 0.01; Student’s *t* test). **(c)** Immunoblots showing the SlPIF4 protein level in WT and *pro* plants at 10 dpi. A same membrane was split into two sheets and incubated with anti-PIF4 and anti-HSP70 antibodies, respectively. Anti-HSP70 was used as a loading control for the western blot analysis. Representative pictures are shown. Relative protein levels are shown on the right side of the blots. Data are presented as the means of three biological replicates (±SD). Asterisks indicate a statistically significant difference from the control in the means (^**^P < 0.01; Student’s *t* test). **(d)** SlPIF4 degradation in cell-free degradation assay. Total proteins extracted from the roots of WT and *pro* plants inoculated with *R. intraradices* were incubated with or without MG132 (a 26S proteasome inhibitor) over the indicated time course, and the protein levels of SlPIF4 were detected using an anti-MBP antibody. Anti-HSP70 was used as a loading control for the western blot analysis. For **(a)**–**(d)**, the plants inoculated with (AM) or without (NM) *R. intraradices* were grown in a sterilized soil: quartz sand: vermiculite mixture (1:1:1) under Pi deficient condition (without KH_2_PO_4_ but with the addition of 1 mM KCl). **(e)**–**(f)** Dual-luciferase assay showing the regulatory effect of SlPIF4 influenced by SlDELLA on the promoters of *SlCCD7* and AMS-specific *PT4*. The ratio of firefly luciferase (LUC) and renilla luciferase (REN) of the empty vector (EV) plus promoter was set at ‘1’. Data are presented as the means of six biological replicates (±SD). Different letters indicate significant differences by one-way ANOVA followed by post *hoc* Tukey test (*P* < 0.05). **(g)**–**(h)** Electrophoresis mobility shift (EMSA) assay. The biotin-labeled *SlCCD7* and AMS-specific *PT4* oligos was used as SlPIF4-targeted DNA sequence. 1×, 3×, and 5× indicated the intensity of the GST-SlDELLA protein, and 1×, 2×, and 3× indicated the intensity of the GST protein. The protein purified from the empty vector was used as a negative control.

Having ascertained that SlDELLA interacts with SlPIF4 genetically and physically, we sought to determine their functional interplay. To test this, we first detected the SLs content of *pro* plants and found that the relative SLs content was lower than that in WT AM plants at 10 dpi (Fig. S11a, see online supplementary material). Additionally, the transcript levels of *SlCCD7*, *SlCCD8,* and *SlMAX1* followed the same trend as the relative content of SLs (Fig. 7a; Fig.  S11c and d, see online supplementary material). In WT plants, the content of phosphorus increased in AM plants in comparison with NM plants at 30 dpi. However, the extent of AMS-induced increase in P content was less pronounced in the *pro* mutant than in WT plants at 30 dpi (Fig. S11b, see online supplementary material), which corresponds with the expression levels of *PT4* and *PT5* (Fig. 7b). These results demonstrate that the biosynthesis of root SLs and Pi uptake are inhibited by SlPIF4 but promoted by SlDELLA.

**Figure 8:**
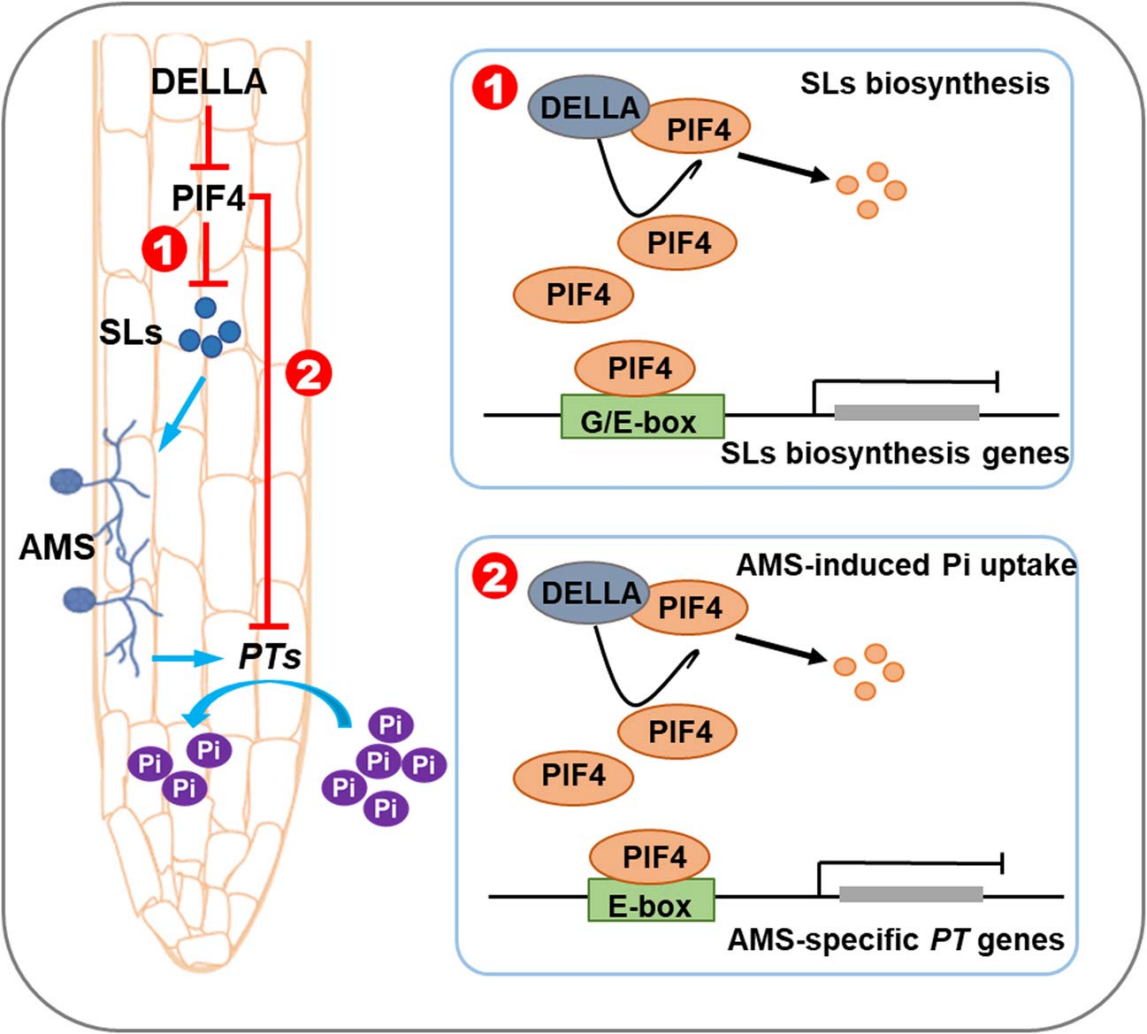
A proposed model showing SlDELLA-SlPIF4-SLs/*PTs* signaling pathway regulates AMS and phosphate uptake in tomato. SlDELLA interacts with SlPIF4 to reduce SlPIF4 protein stability and its transcriptional activity toward downstream target genes, including SLs biosynthesis genes and AMS-specific phosphate transporter genes, thus promoting SLs accumulation, AMS development, and Pi uptake in tomato.

To further explore the molecular mechanism underlying the influence of SlDELLA on SlPIF4 protein at a molecular level, we first asked whether SlDELLA might regulate the transcript level of *SlPIF4* by using the WT and *pro* plants. The results indicated that the *SlPIF4* transcript level between WT and *pro* plants remained almost unchanged (Fig. S11e, see online supplementary material). In contrast, the SlPIF4 protein abundance was sharply increased in *pro* plants in comparison with WT plants according to the immunoblot analysis (Fig. 7c), indicating that SlDELLA affected SlPIF4 protein accumulation by reducing its stability. Cell-free degradation assay was carried out to further confirm the result. The total protein extracted from WT and *pro* plants was incubated with equal amounts of purified recombinant MBP-SlPIF4 protein, respectively. The results showed that MBP-SlPIF4 was gradually degraded with the extension of the ATP application time. However, the MBP-SlPIF4 protein degradation rate was slower with *pro*-extracted protein than that with WT-extracted protein (Fig. 7d; Fig.  S11f, see online supplementary material). Additionally, we performed dual-luciferase assay to verify that SlDELLA influences the transcriptional activity of SlPIF4. When SlPIF4 and SlDELLA co-expressed with the gene promoters, there was no change in compared with the control (Fig. 7e and f; Fig.  S12a–c, see online supplementary material). To further substantiate the results, we carried out EMSA assay using recombinant protein and probes. The results indicated that the bound probes were reduced constantly along with the increase GST-SlDELLA, but not the GST (Fig. 7g and h; Fig.  S12d–f, see online supplementary material). Collectively, these data indicated that SlDELLA reduced the SlPIF4 protein stability and attenuated its transcriptional activity.

## Discussion

Arbuscular mycorrhizal symbiosis is precisely controlled by many transcription factors from the pre-signals exchange to the final arbuscule formation and degradation [48, 49]. Although the role of many transcription factors has been revealed, such as GRAS family and AP2/ERF family, the underlying molecular mechanisms of other TFs regulating AMS are not well elucidated [50, 51]. Previous studies indicated that light, especially high R/FR light significantly promoted AMF colonization in tomato and *L. japonicas* [17]. Our recent study also pointed out that a phyB–HY5–SLs cascade signaling facilitated the AMS [18]. Additionally, the transcript level of *PIF3* decreased in roots and leaves of trifoliate orange inoculated with AMF (*Funneliformis mosseae*) [52, 53]. Here, we revealed the transcript and protein abundance of SlPIF4 significantly decreased in mycorrhizal tomato roots (Fig. 1a and b). Further genetic analysis showed that *SlPIF4* negatively regulates AMF colonization (Fig. 1c–e; Fig.  S3, see online supplementary material).

The DELLA proteins have central functions in modulating AM development. On the one hand, the deficiency of DELLA resulted in a significant decrease in mycorrhizal colonization in *M. truncatula*, *O. sativa,* and pea [34, 35]. Consistent with this, we found that SlDELLA positively regulated the AMS in tomato. The hyphae, arbuscules, and vesicles in *pro* roots were less than in WT plants, as well as the transcript levels of *BCP1* (Fig. S5a–c, see online supplementary material). On the other hand, DELLA can also interact with other transcription factors to influence the AMS, such as CYCLOPS, NSP2, and MYB1 [36, 37, 54]. Additionally, DELLA interacted with PIFs to promote its degradation or sequestrating it from targeted genes in *Arabidopsis* [38, 39]. Here, we also found SlDELLA interacted with SlPIF4 physically by Y2H, BiFC, SLCA, and pull-down assays (Fig. 2a–e). Surprisingly, both SlPIF4 and SlDELLA regulated the SLs biosynthesis and AMS-induced Pi uptake, and also the transcript levels of SLs biosynthesis genes and AMS-specific *PTs*, but acted opposite roles (Figs 4, 6, 7a and b; Figs  S8a–d and S11a–d, see online supplementary material). NSP1 and NSP2, the GRAS-type transcriptional regulators, not only regulated SLs biosynthesis controlling the expression of *D27* and *MAX1* in *M. truncatula* and rice, but also were required for AMF infection, and the SLs and mycorrhization levels decreased in double *nsp1*/*nsp2* mutants [55]. Additionally, DELLAs could form a protein complex with NSP2-NSP1 and other proteins to influence mycorrhizal symbiosis [35, 37, 54]. Here, we also showed that SlDELLA interacted with SlPIF4 to regulate the SLs biosynthesis to control the AMS in tomato; however, the relationship between NSP2-NSP1 and DELLA-PIF4 in the regulation of AMS in tomato needs further study.

Previous studies have shown that PIFs bind to G/E-box motifs (CANNTG) to regulate target gene transcriptionally [25]. Our previous study indicated that PIF4 directly regulated the expression of *DELLA* in response to cold stress [24]. PIF4 and PIF5 also regulated the expression of *IAA19* and *IAA29* by binding to the G-box (CACGTG) motifs in their promoters to participate in auxin-mediated hypocotyl phototropic growth [56]. PIF1 directly regulated *PORC* in a G-box-dependent manner to control chlorophyll biosynthesis in *Arabidopsis* [57]. Consistently, our study showed that SlPIF4 suppressed the transcription of SLs biosynthesis genes and AMS-specific *PTs* by binding to G/E-box motifs of their promoters using dual-luciferase, ChIP-qPCR, and EMSA assays (Figs. 5a–c and  6e–g). Additionally, SlPIF4 activity is tightly controlled by SlDELLA. There was a significant difference in SlPIF4 protein abundance rather than *SlPIF4* transcript between WT and *pro* plants (Fig. 7c; Fig.  S11e, see online supplementary material). We also found that SlDELLA enhanced the degradation rate of SlPIF4 by cell-free protein degradation assay (Fig. 7d). Besides, SlDELLA inhibited the transcription of SlPIF4 to its downstream genes (Fig. 7e–h; Fig.  S12, see online supplementary material). Consistent with this, the sequestration and degradation of PIF3 by DELLAs contribute to a reduction of PIF3 binding ability to its target genes in *Arabidopsis* [38].

AMS are well known for their role in promoting mineral nutrition acquisition of plants, especially phosphorus, and the symbiotic route is responsible for about 70% of the overall Pi delivery [58, 59]. Obviously, in the study, SlPIF4 negatively regulates the SLs biosynthesis to control AMS, thus influencing the phosphate uptake (Fig. 8). Except that, SlPIF4 also inhibited the transcription of AMS-specific *PTs*. In *M. truncatula*, *PHT1;4* (*PT4*), the AMS-specific Pi transporter gene, was mutated, which not only impaired the development of the interaction but also inhibited the symbiotic Pi uptake [60]. Consistent with this, mutations in either *PT11* or *PT13* in *O. sativa* affected the development of symbiosis [59, 61]. In tomato, we found that the *PT11* ortholog *PT4* and *PT5* in two *pif4* mutants were increased significantly compared with WT plants, which was the opposite in *PIF4*#89 plants (Fig. 6c and d). More importantly, the deletion of *SlPIF4* significantly improved the AMS-induced Pi uptake (Fig. 6a), and the AMS-induced P content was lower in *PIF4*#89 plants than in WT plants (Fig. 6b). Except for symbiotic Pi uptake, plants can also absorb Pi directly, in which the phosphate transporters PHT1 family genes are most relevant for Pi uptake from soil [62]. Previous studies reported that PIF4 and PIF5 inhibited the Pi uptake and accumulation directly by negatively modulating the expression of Pi uptake and Pi starvation-responsive genes in *A. thaliana* [63, 64]. Here, we also found that the total P contents in *pif4*#12 and *pif4*#14 plants were higher than in WT plants inoculated without *R. intraradices* (Fig. 6a), which indicated that SlPIF4 may regulate Pi uptake under Pi deficient conditions in an AMS independent pathway. However, whether and which PHT1 family genes are involved remains to be further verified in tomato.

In conclusion, our findings revealed a novel SlDELLA-SlPIF4-SLs/*PTs* signaling pathway that regulates AMF colonization and Pi uptake in tomato. SlDELLA interacted with SlPIF4 and positively regulated AMF colonization, which was contrary to the regulation of SlPIF4 to AMS. As a transcription factor, SlPIF4 regulates SLs biosynthesis and AMS-induced phosphate uptake by binding to the G/E box motifs of the SLs biosynthesis genes and AMS-specific *PTs* promoters directly. SlDELLA improves the transcript levels of SlPIF4 target genes by reducing protein stability and attenuating the SlPIF4 transcriptional activity (Fig. 8). Our finding reveals a regulatory mechanism of SlDELLA-SlPIF4 module in AMS. We clarify a molecular basis that SlPIF4 interacts with SLs signaling to regulate the AMS and provide a potential way to improve phosphate utilization efficiency by targeting the AMS-specific phosphate transporter genes *PTs*.

## Materials and methods

### Plant materials, growth conditions, and chemical treatments

The wild-type (WT, *Solanum lycopersicum* cv Ailsa Craig) and *procera* (*pro*) mutant, a putative DELLA mutant, were used in this study. The *SlPIF4*-overexpressing and CRISPR/Cas9 *SlPIF4* mutants plants were generated as the previous description [24]. The VIGS technique was used to generate *SlDELLA* silencing tomato plants. The target cDNA of *SlDELLA* was amplified using the specific primers presented in Table S1 (see online supplementary material), and then inserted into TRV2. VIGS was conducted as the previous description [65].

The VIGS plants were grown in the chambers at 21°C (day) and 20°C (night), with a photoperiod of 12 h/12 h (day/night) and a light intensity 200 μmol m^−2^ s^−1^. Other tomato plants were grown in the chambers at 25°C (day) and 20°C (night) with the same photoperiod and light intensity. The mycorrhizal inoculation method was conducted as the previous description [66], and the AMF species *R. intraradices* UT126a [15] with a mixture of quartz sand and vermiculite (1:1, v/v) was used in this study. 4-leaf stage seedlings were grown in a 600 mL pot. One plant per pot inoculated with ~400 *R. intraradices* spores. Using Hoagland’s nutrient solution (1 mM KCl replaces KH_2_PO_4_) the pot plants were watered three times a week. For the chemical treatments, tomato seedlings were treated with l μM *rac*-GR24 (CR9420; Coolabel (Beijing, China)) by application to the soil three times a week. Seedlings treated with ddH_2_O served as control. The first application started at 3 dpi (days post-inoculation).

### Morphological analyses and microscopy

Mycorrhizal colonization was visualized using fluorescence imaging, following established procedures [67]. Briefly, segments of mycorrhizal roots were immersed in 50% ethanol (v/v) for over 4 hours, and subsequently acidified with 0.1 M HCl for approximately 2 hours following incubation in 20% KOH (w/v) for about 3 d. The roots were then rinsed and submerged in a staining solution of WGA-Alexa Fluor 488 (1 μg mL^−1^ in PBS) in the dark for 12 h. Imaging was observed using a laser scanning confocal microscope (Leica TCS SL, Leica Microsystems (Heidelberg, Germany)) with standard settings for WGA-Alexa Fluor 488.

For quantification of root length colonization (RLC), the roots inoculated with *R. intraradices* were treated under 95°C as follows: 10% KOH (w/v) for 35 min, 2% HCl for 5 min; ddH_2_O for rinsing three times, trypan blue (0.05%) for staining at 95°C for 10 min, and lactic acid mixed with glycerin (1:1, v/v) used for bleaching for 3 days. Images were taken using Leica DM4000B microscope (Leica Microsystems (Heidelberg, Germany)). Per treatment contains 6–12 plants and about 300 root segments were placed on a slide with a 0.5 cm gridline to assess the RLC according to the previous description [68]. The RLC was calculated according to the following formula: (hyphae, arbuscules, vesicles, and hyphopodia)/intersections of root segment and grid. The size of the grid is 0.5 cm [15].

### Measurements of strigolactones content

The measurement of SLs in the roots was performed as the previous description [66] with some modifications. Homogenized 0.5 g roots samples with 40% acetone (v/v) and the supernatant removed after centrifugation at 4°C. Next, extracted with 500 mL 50% acetone (v/v) and collected the supernatant after centrifugation. Subsequently, samples were evaporated under a vacuum to 1 mL and then extracted with ethylacetate and vortexed centrifugation. A total of 750 mL organic phase was evaporated under vacuum to dry, and redissolved with 25% acetonitrile (1:3 v/v). The SLs content was analysed by LC–MS/MS.

The extraction of root exudates SLs was consulted [69] and made some necessary changes. Four-leaf stage seedlings were cultured under P deficient (-P; 0.95 mM KCl replaces 0.05 mM KH_2_PO_4_) condition for 10 days. The root exudates were collected after 6 hours. The exudates (50 mL) were passed through the CNWBOND HC-C18 SPE cartridge (SBEQ-CA0854; CNW (Shanghai, China)) pre-equilibrated with methanol. Subsequently, root exudates were collected with 100% acetone after washing with ddH_2_O. Samples were evaporated under a vacuum to 1 mL, then extracted with ethylacetate and subjected to vortex centrifugation. A total of 750 mL organic phase was evaporated under a vacuum to dry and redissolved with 25% acetonitrile (1:3 v/v). The root exudates SLs were analysed by LC–MS/MS.

### Measurements of P content

The P measurement was conducted according to the previous description [70]. Briefly, the total plants were harvested and heated or steamed to de-enzyme at 105°C for 30 min and dried at 65°C for 3 days. The 0.1 g samples were weighed and digested with H_2_SO_4_ at 150°C. For redox reaction, 30% H_2_O_2_ was added and the homogenates diluted to 50 mL (V_2_) using ddH_2_O after filtration. The 5 mL homogenates were made up to 45 mL with ddH_2_O and adjusted the pH (7 ~ 8) using 6 M NaOH and 2 M H_2_SO_4_, then mixed with 5 mL assay solution (V_1_, 4.5 M H_2_SO_4_, 0.5% antimony potassium tartrate, 85 mM L-ascorbic acid and 1% NH_4_MoO_4_), and reacted at 28°C for 30 min (V_3_ = 50 mL). The P content was calculated based on the absorbance at 700 nm: Wp = (*c* × V_3_ × (V_2_/V_1_) × 10^−3^)/m.

### RNA isolation and quantitative real-time PCR (RT-qPCR) analysis

RNA extraction, reverse transcription, and real-time PCR assay were performed according to the previous description [15]. The samples were extracted from tomato roots. The relative expression was calculated by the 2^-ΔΔC*T*^ method [71]. The *ACTIN2* was a reference gene for normalization. Primers are presented in Table S2 (see online supplementary material).

### Protein extraction and immunoblot analysis

Tomato roots were homogenized in protein extraction buffer consisting of Tris–HCl (100 mM, pH = 8.0), NaCl (100 mM), EDTA (1 mM), Triton X-100 (1% (v/v)), phenylmethylsulfonyl fluoride (1 mM), dithiothreitol (0.2%) and protease inhibitor cocktail. The supernatants with 2× loading buffer were separated by SDS-PAGE after vortices and centrifugation at 4°C. Anti-PIF4 (AS163955; Agrisera (Vännäs, Sweden)), anti-DELLA (Hangzhou Biospring Tech. Co. Ltd, catalog no. BSM2310005 (Hangzhou, China)), anti-HSP70 (Beijing BGI Protein R&D Center Co., Ltd (Beijing, China)) and anti-rabbit antibody (Cell Signaling Technology (Boston, MA, USA)) were used in this study.

### Recombinant proteins and electrophoretic mobility shift (EMSA) assay

The MBP-SlPIF4 was constructed by ligating the CDS region of SlPIF4 into the MBP vector by homologous recombination and heat shocked into *Escherichia coli* strain BL21 (DE3). The 0.1 mM isopropyl β-D-1-thiogalactopyranoside (IPTG) was used to induce the MBP-SlPIF4 protein for 12 h. Amylose Resin High Flow was used to purify the MBP-SlPIF4 protein. The *SlCCD7*, *SlCCD8*, *SlMAX1*, *PT4,* and *PT5* promoter probes containing a G/E-box motif and the mutant probes were labeled by biotin using the Biotin 3′ End DNA Labeling Kit (catalog. No. 89818; Pierce (Rockford, IL, USA)). The labeled and unlabeled oligonucleotides were annealed to double-stranded DNA probes. The annealed and unlabeled oligonucleotides were used as competitor probes. EMSA assay was carried out by the LightShift Chemiluminescent EMSA kit (catalog. no. 20148; Thermo Fisher Scientific (Waltham, MA, USA)). The primers are listed in Table S3 (see online supplementary material).

### Chromatin immunoprecipitation (ChIP) quantitative PCR assay

Chromatin immunoprecipitation (ChIP) was carried out according to the instructions of the EpiQuikTM Plant ChIP Kit (P-2014; Epigentek (Farmingdale, NY, USA)). The root samples were harvested from the *SlPIF4* overexpressing (*PIF4*#89) or WT plants inoculated with *R. intraradices* at 10 dpi under Pi deficient condition. Anti-HA antibody (26 183; Pierce (Rockford, IL, USA)) and anti-mouse antibody (AP124P; Millipore (Billerica, MA, USA)) were used to immunoprecipitate the chromatin. The primers are listed in Table S4 (see online supplementary material).

### Dual-luciferase transient expression (LUC) assay in *N. benthamiana*

The SK-SlPIF4 was constructed by ligating the CDS region of SlPIF4 into the pGreenII_0029_62-SK vector using the primer sequences presented in Table S5 (see online supplementary material). The LUC-*SlCCD7*, LUC-*SlCCD8*, LUC-*SlMAX1*, LUC-*PT4,* and LUC-*PT5* were constructed by ligating the promoters of *SlCCD7*, *SlCCD8*, *SlMAX1*, *PT4*, and *PT5* into the pGreen II 0800-LUC vector, respectively. The constructs were electroporated into *Agrobacterium tumefaciens* strain GV3101: psoup, respectively. The infiltration buffer with an OD _600_ = 0.75, consists of 10 mM MES, 150 μM acetosyringone, and 10 mM MgCl2 (pH 5.6). The TFs mixed with promoters (10:1, v/v) were infiltrated into the leaves of *N. benthamian*. The LUC/REN activities were detected by Dual-Luciferase Reporter Assay Kit (DL101–01; Vazyme) after 3 days.

### Bimolecular fluorescence complementation (BiFC) assay

The YFP^n^-SlPIF4 and YFP^c^-SlDELLA were constructed by ligating the CDS regions of SlPIF4 and SlDELLA into the N-terminal of YFP (YFP^n^) and the C-terminal of YFP (YFP^c^) vectors using the primer sequences listed in Table S6 (see online supplementary material). *A. tumefaciens* strain GV3101 containing YFP^n^/ YFP^c^-SlDELLA, YFP^c^/ YFP^n^-SlPIF4, YFP^n^-SlPIF4/ YFP^c^-SlDELLA and YFP^n^/YFP^c^ was rinsed with infiltration buffer (10 mM MES, 150 μM acetosyringone, and 10 mM MgCl_2_ (pH 5.6)) and adjusted OD _600_ = 0.75 and the bacterium was infiltrated into *N. benthamiana* leaves, respectively. Fluorescence was observed using an A1 confocal laser scanning microscope (Nikon, Tokyo, Japan) after 48 hours.

### Split luciferase complementation assay

The cLUC-SlPIF4 was constructed by ligating the CDS region of SlPIF4 into the pCAMBIA-cLUC. The nLUC-SlDELLA was constructed by ligating the CDS region of SlDELLA into the pCAMBIA-nLUC vectors. *A. tumefaciens* strain GV3101 containing nLUC/ cLUC, cLUC/ nLUC-SlDELLA, cLUC-SlPIF4/ nLUC, and cLUC-SlPIF4/nLUC-SlDELLA was rinsed with infiltration buffer (10 mM MES, 150 μM acetosyringone, and 10 mM MgCl_2_ (pH 5.6)) and adjusted OD _600_ = 0.75. The bacterium was infiltrated into *N. benthamiana* leaves for 2 days, respectively. Then 1 mM D-luciferin was brushed on the infiltrated leaves and left in darkness for 10 min. The signals were detected using an HRPCS5 camera (Photek (Lancaster, PA, USA)). The leaf disks were incubated with 100 μL of 1 mM luciferin solution and left in darkness for 10 min. The luciferase activity was detected using the Centro LB 960 Microplate Luminometer (Berthold Technologies (Wildbad, Germany)). The primers are presented in Table S7 (see online supplementary material).

### Yeast two-hybrid (Y2H) assay

The AD-SlPIF4 was constructed by ligating the CDS region of SlPIF4 into the pGADT7 vector by homologous recombination. The BD-SlDELLA was constructed by ligating the CDS region of SlDELLA into the pGBKT7 vectors by homologous recombination. The primers are presented in Table S8 (see online supplementary material). The recombined constructs were transformed into the yeast strain Y2HGold and then cultured on SD medium (−Leu/−Trp) for 2 days or SD medium (−Leu/−Trp/−Ade/-His) medium for 5 days at 30°C.

### *In vitro* pull-down assay

The MBP-SlPIF4 and GST-SlDELLA were constructed by ligating the CDS regions of *SlPIF4* or *SlDELLA* into the MBP or GST vectors by homologous recombination using the primer sequences presented in Table S9 (see online supplementary material), respectively. MBP and MBP-SlPIF4 fusion proteins were extracted with extraction buffer [Tris–HCl (1 M, pH = 8.0), EDTA (0.5 M, pH = 8.0), NaCl (5 M), and Triton X-100] and kept immobilized on Amylose Resin (NEB). GST-SlDELLA was purified using Pierce Glutathione Agarose (Thermo Fisher Scientific (Waltham, MA, USA)). Amylose Resin (NEB) containing MBP or MBP-SlPIF4 was incubated with GST-SlDELLA at 4°C for 4 hours using the SDS-PAGE to separate the pulled-down proteins after washing. Using anti-GST antibody, anti-MBP antibody (Cell Signaling Technology (Boston, MA, USA)), and anti-mouse antibody (AP124P; Millipore (Billerica, MA, USA)) to determine the protein.

### Cell-free protein degradation assay

The protein extraction buffer consists of NaCl (10 mM), DTT (5 mM), MgCl_2_ (10 mM), Tris–HCl (25 mM, pH = 5.6), phenylmethylsulfonyl fluoride (1 mM), and ATP (1 mM). The total proteins from the roots of WT and *pro* plants were extracted using the extraction buffer. The total protein extracts were incubated with recombinant MBP-SlPIF4 protein for 0, 7, 14, 21, and 28 min, and SDS-PAGE assay was performed. Antinti-MBP antibody (Cell Signaling Technology (Boston, MA, USA)) was used to determine the recombinant protein. Anti-mouse IgG antibody (AP124P; Millipore (Billerica, MA, USA)) was used as the second antibody.

### Statistical analysis

GraphPad Prism 9.0 Software was used for analysing the data. Protein quantification was fulfilled with ImageJ. SPSS statistical software was used to conduct the statistical analysis. One-way ANOVA followed by Tukey’s test or two-tailed Student’s *t-*test was performed. For each experiment, at least three biological replicates were used. The samples were taken from two different plant roots as a biological replicate.

### Accession numbers

Sequences can be acquired at https://solgenomics.net, SL4.0: *ACTIN2* (Solyc03g078400), *PIF1a* (Solyc09g063010), *PIF1b* (Solyc06g008030), *PIF3* (Solyc01g102300), *PIF4* (Solyc07g043580), PIF7a (Solyc03g115540), PIF7b (Solyc06g069600), *PIF8a* (Solyc01g090790), *PIF8b* (Solyc01g018510), *BCP1* (Solyc10g081520), *PT4* (Solyc06g051850), *PT5* (Solyc06g051860), *CCD7* (Solyc01g090660), *CCD8* (Solyc08g066650), *MAX1* (Solyc08g062950), *DELLA* (Solyc11g01126).

## Supplementary Material

Web_Material_uhae195

## Data Availability

All data in this article are available within the article and its online supplementary data.
