# Supplementary material for: SlDELLA interacts with SlPIF4 to regulate arbuscular mycorrhizal symbiosis and phosphate uptake in tomato
Source: Hortic Res. 2024 Jul 21;11(9):uhae195. doi: 10.1093/hr/uhae195 (PMC11384114; doi:10.1093/hr/uhae195)
Supplement: Web_Material_uhae195 [file web_material_uhae195.zip › Supplementary figures.doc]

**Article title: SlDELLA interacts with** **SlPIF4 to** **regulate arbuscular mycorrhizal symbiosis and phosphate uptake in tomato**

**Authors:** Lan Li^1^, Shibei Ge^1,4^, Liqun He^1^, Ruicheng Liu^1,2^, Yuhong Mei^1^, Xiaojian Xia^1,3^, Jingquan Yu^1,3^ and Yanhong Zhou^1,2,3*^

The following Supporting Information is available for this article:

**Fig S1.** The transcript levels of eight *SlPIFs* and AMS-marker genes in the roots of WT plants at 10 dpi.

**Fig S2**. Time-course analysis of root length colonization in mycorrhizal roots of WT plants.

**Fig S3.** *SlPIF4* negatively regulates the arbuscular mycorrhizal symbiosis in tomato.

**Fig S4.** Relative gene expression and the protein abundance of SlDELLA.

**Fig S5.** *SlDELLA* positively regulates the arbuscular mycorrhizal symbiosis in tomato.

**Fig S6.** Efficiency of *SlDELLA* gene silencing.

**Fig S7.** *SlPIF4* negatively regulates the biosynthesis and exudation of SLs in tomato*.*

**Fig S8.** *SlPIF4*-mediated AMS is partly dependent on SLs biosynthesis and accumulation.

**Fig S9.** The schematic diagram of *SlCCD7*, *SlCCD8* and *SlMAX1*promoters and the recombination vectors.

**Fig S10.** The schematic diagram of AMS-specific *PT4* and *PT5* promoters and the recombination vectors.

**Fig S11.** SlDELLA influences the stability of SlPIF4 and the expressiom of its target genes.

**Fig S12.** SlPIF4 inhibits the transcription of strigolactone biosynthesis genes and AMS-specific *PTs*, which is attenuated by SlIDELLA.

**Fig S1.** **The transcript levels of eight *SlPIFs* and AMS-marker genes in the roots of WT plants at 10 dp.** The plants were grown in a sterilized soil: quartz sand: vermiculite mixture (1:1:1) inoculating with (AM) or without (NM) *R. intraradices* under Pi deficient condition (without KH_2_PO_4_ but with the addition of 1 mM KCl). Data are presented as the means of four biological replicates in (a) and three biological replicates in (b) (±SD). Asterisks indicate a statistically significant difference from the control in the means (**P* < 0.05; ***P* < 0.01; Student’s *t* test).

**Fig S2.** T**ime-course analysis of root length colonization in mycorrhizal roots of WT plants.**The plants were grown in a sterilized soil: quartz sand: vermiculite mixture (1:1:1) inoculating with (AM) or without (NM) *R. intraradices* under Pi deficient condition (without KH_2_PO_4_ but with the addition of 1 mM KCl). Data are presented as the means of six biological replicates (±SD). Asterisks indicate a statistically significant difference from the control in the means (***P* < 0.01; Student’s *t* test).

**Fig S3.** ***SlPIF4* negatively regulates the arbuscular mycorrhizal symbiosis in tomato.** (a) Representative images of WGA-AF488-stained roots of WT and *SlPIF4* overexpressing (*PIF4*#89) plants at 20 dpi. BF, bright-field image. Merge, merged WGA-AF488 and BF image. Scale bar = 100 µm. (b) The root length colonization of the roots in WT and *SlPIF4* overexpressing plants at 20 dpi. Each replication contains 80 root segments of two plants. Data are presented as the means of four biological replicates (±SD). Asterisks indicate a statistically significant difference from the control in the means (***P* < 0.01; Student’s *t* test). (c) Transcripts of AMS-marker gene *BCP1* in roots of WT and *SlPIF4* overexpressing (*PIF4*#89) plants at 20 dpi. Data are presented as the means of four biological replicates (±SD). Asterisks indicate a statistically significant difference from the control in the means (***P* < 0.01; Student’s *t* test). (d) Frequency graphs showing the distribution of arbuscule size classes (cross section area; μm2) in arbuscule populations measured in WT, *pif4* mutants and *SlPIF4* overexpressing plants with *R. irregularis* at 20 dpi. WT was set as the control. (e) Images of WGA-AF488 stained arbuscule of the AM roots at 20 dpi. BF, bright-field image. Merge, merged WGA-AF488 and BF image. Scale bar, 5 μm. The WT, *pif4* mutants and *SlPIF4* overexpressing (*PIF4*#89) plants were grown in a sterilized soil: quartz sand: vermiculite mixture (1:1:1) inoculating with (AM) or without (NM) *R.* *intraradices* under Pi deficient condition (without KH_2_PO_4_ but with the addition of 1 mM KCl).

**Fig S4. Relative gene expression and the protein abundance of SlDELLA.** (a) The relative expression of *SlDELLA* in the roots of WT plants at 10 dpi. Data are presented as the means of four biological replicates (±SD). ns: no significant difference from the control in the means (*P* > 0.05; Student’s *t* test). (b-c) Immunoblots showing the SlDELLA protein level in roots of WT plants at 10 dpi. A same membrane was split into two sheets and incubated with anti-DELLA and anti-HSP70 antibodies, respectively. Representative pictures are shown in (b). The relative protein levels are shown in (c), and the relative protein levels of WT in NM roots were set to 1.00. Data are presented as the means of three biological replicates (±SD). Asterisks indicate a statistically significant difference from the control in the means (***P* < 0.01; Student’s *t* test).

**Fig S5.** ***SlDELLA* positively regulates the arbuscular mycorrhizal symbiosis in tomato.** (a) Representative images of WGA-AF488-stained roots of WT and *pro* plants at 20 dpi. BF, bright-field image. Merge, merged WGA-AF488 and BF image. Scale bar = 100 µm. (b) The root length colonization of hyphae, arbuscule and vesicles in roots of of WT and *pro* plants at 20 dpi. Each replication contains 80 root segments of two plants. Data are presented as the means of four biological replicates (±SD). Asterisks indicate a statistically significant difference from the control in the means (***P* < 0.01; Student’s *t* test). (c) Transcripts of AMS-marker gene *BCP1* in roots of WT and *pro* plants at 20 dpi. Data are presented as the means of four biological replicates (±SD). Asterisks indicate a statistically significant difference from the control in the means (***P* < 0.01; Student’s *t* test). The WT and *pro* plants were grown in a sterilized soil: quartz sand: vermiculite mixture (1:1:1) inoculating with (AM) or without (NM) *R. intraradices* under Pi deficient condition (without KH_2_PO_4_ but with the addition of 1 mM KCl).

**Fig S6.** **Efficiency of *SlDELLA* gene silencing.** (a) Expression of *SlDELLA* in WT-pTRV-*SlDELLA*) *pif4*#12- pTRV-*SlDELLA* plants. The gene expression level was determined relative to that in the WT-pTRV plants without (NM) *R. intraradices* (set at a value of 1) by RT-qPCR. Data are presented as the means of six biological replicates (±SD). Asterisks indicate a statistically significant difference from the control in the means (***P* < 0.01; Student’s *t* test).

**Fig S7. *SlPIF4* negatively regulates the biosynthesis and exudation of SLs in tomato.** (a) Relative contents of SLs from root exudates of WT and *pif4* mutant plants. The plants were grown under Pi sufficient (+Pi; 1 mM KH_2_PO_4_) or Pi deficient (-Pi; 0.05 mM KH_2_PO_4_ with the addition of 0.95 mM KCl) conditions for 10 days following by collecting root exudates for 6 hours. Data are presented as the means of four biological replicates (±SD). Different letters indicate significant differences by One-way ANOVA followed by post *hoc* Tukey test (*P* < 0.05). (b-d) Transcripts of strigolactones biosynthesis genes in roots of WT and *pif4* mutant plants under Pi sufficient (+Pi; 1 mM KH_2_PO_4_) or Pi deficient (-Pi; 0.05 mM KH_2_PO_4_ with the addition of 0.95 mM KCl) conditions for 10 days. Data are presented as the means of four biological replicates (±SD). Different letters indicate significant differences by One-way ANOVA followed by post *hoc* Tukey test (*P* < 0.05).

**Fig S8. *SlPIF4*-mediated AMS is partly dependent on SLs biosynthesis and accumulation.** (a) Relative contents of SLs from WT and *SlPIF4* overexpressing (*PIF4*#89) plants roots at 10 dpi. Data are presented as the means of three biological replicates (±SD). Different letters indicate significant differences by One-way ANOVA followed by post *hoc* Tukey test (*P* < 0.05). (b-d) Transcripts of strigolactones biosynthesis genes in the roots of WT and *SlPIF4* overexpressing (*PIF4*#89) plants at 10 dpi. Data are presented as the means of four biological replicates (±SD). Different letters indicate significant differences by One-way ANOVA followed by post hoc Tukey test (*P* < 0.05). (e-f) Transcripts of *PT4* and *PT5* in roots of WT and *SlPIF4* overexpressing (*PIF4*#89) plants applied with or without 1 μM *rac*-GR24 at 20 dpi. Data are presented as the means of four biological replicates (±SD). Different letters indicate significant differences by One-way ANOVA followed by post hoc Tukey test (*P* < 0.05). For (a-d), the plants inoculated with (AM) or without (NM) *R.* *intraradices* were grown in a sterilized soil: quartz sand: vermiculite mixture (1:1:1) under Pi deficient condition (without KH_2_PO_4_ but with the addition of 1 mM KCl). For (e-f), the WT and *SlPIF4*-overexpressing (*PIF4*#89) plants inoculated with *R.* *intraradices* were treated with ddH_2_O (Control) or + *rac*-GR24 (1 μM) three times a week.

**Fig S9.** **The schematic diagram of *SlCCD7*, *SlCCD8* and *SlMAX1*promoters and the recombination vectors.** (a) Schematic diagram of the *SlCCD7*, *SlCCD8* and *MAX1*. The promoters of *SlCCD7*, *SlCCD8*, and *SlMAX1* contain the E-box (CANNTG) or G-box (CACGTG) within 3000bp upstream of the genes. The E-box motif was marked in green. The G-box motif was marked in pink. Oligonucleotide of genes were used in the EMSA assay. The G/E-box sequences were marked in red. The LUC sequences were blue. The ChIP-qPCR sequences were orange.

**Fig S10. The schematic diagram of AMS-specific *PT4* and *PT5* promoters and the recombination vectors.** (a) Schematic diagram of the *PT4* and *PT5* promoters*.* The promoters of *PT4* and *PT5* contain the E-box (CANNTG) within 3000bp upstream of the genes. The E-box motif was marked in green. Oligonucleotide of genes were used in the EMSA assay. The E-box sequences were marked in red. The LUC sequences were blue. The ChIP-qPCR sequences were orange.

**Fig S11. SlDELLA influences the stability of SlPIF4 and the expressiom of its target genes.** (a) Relative contents of SLs from WT and *pro* plants roots at 10 dpi. Data are presented as the means of three biological replicates (±SD). Different letters indicate significant differences by One-way ANOVA followed by post *hoc* Tukey test (*P* < 0.05). (b) Total P concent of WT and *pro* plants at 30 dpi. Data are presented as the means of four biological replicates (±SD). Different letters indicate significant differences by One-way ANOVA followed by post *hoc* Tukey test (*P* < 0.05). (c-d) Transcripts of *SlCCD8* and *SlMAX1* in the roots of WT and *pro* plants at 10 dpi. Data are presented as the means of four biological replicates (±SD). Different letters indicate significant differences by One-way ANOVA followed by post *hoc* Tukey test (*P* < 0.05). (e) Transcripts of *SlPIF4* in WT and *pro* plants roots at 10 dpi. Data are presented as the means of three biological replicates (±SD). ns: no significant difference from the control in the means (*P* > 0.05; Student’s *t* test). (f) SlIF4 degradation in cell-free degradation assay. Total proteins extracted from the WT and *pro* inoculated with *R. intraradices*, were incubated with or without MG132 (a 26S proteasome inhibitor) over the indicated time course, and the protein levels of SlPIF4 were detected using an anti-MBP antibody. The relative protein levels at 0 min were set to 1.00. Data are presented as the means of three biological replicates (±SD). Asterisks indicate a statistically significant difference from the control in the means (**P* < 0.05; ***P* < 0.01; Student’s *t* test). The plants were grown in a sterilized soil: quartz sand: vermiculite mixture (1:1:1) inoculated with (AM) or without (NM) *R. intraradices* under Pi deficient condition (without KH_2_PO_4_ but with the addition of 1 mM KCl).

**Fig S12. SlPIF4 inhibits the transcription of strigolactone biosynthesis genes and AMS-specific *PTs*, which is attenuated by SlIDELLA.** (a-c) Dual-luciferase assay showing regulatory effects of SlPIF4 influenced by SlDELLA on the promoters *SlCCD8*, *SlMAX1* and *PT5*. The LUC/REN ratios of the empty vector (EV) plus promoter were set at “1. Data are presented as the means of six biological replicates (±SD). Different letters indicate significant differences by One-way ANOVA followed by post *hoc* Tukey test (*P* < 0.05). (d-f) Electrophoresis mobility shift (EMSA) assay. The biotin-labeled *SlCCD8*, *SlMAX1* and *PT5* oligos were used as SlPIF4-targeted DNA sequence. 1×, 3× and 5× indicated the intensity of the GST-SlDELLA protein, and 1×, 2× and 3× indicated the intensity of the GST protein. The protein purified from the empty vector was used as a negative control.
