# Supplementary material for: SlDELLA interacts with SlPIF4 to regulate arbuscular mycorrhizal symbiosis and phosphate uptake in tomato
Source: Hortic Res. 2024 Jul 21;11(9):uhae195. doi: 10.1093/hr/uhae195 (PMC11384114; doi:10.1093/hr/uhae195)
Supplement: Web_Material_uhae195 [file web_material_uhae195.zip › Supplementary tables.doc]

**Article title: SlDELLA interacts with** **SlPIF4 to** **regulate arbuscular mycorrhizal symbiosis and phosphate uptake in tomato**

**Authors:** Lan Li^1^, Shibei Ge^1,4^, Liqun He^1^, Ruicheng Liu^1,2^, Yuhong Mei^1^, Xiaojian Xia^1,3^, Jingquan Yu^1,3^ and Yanhong Zhou^1,2,3*^

The following Supporting Information is available for this article:

**Table S1.** Primers used in VIGS.

**Table S2.** Primers used in RT-qPCR.

**Table S3.** Primers used in EMSA assay.

**Table S4.** Primers used in ChIP-qPCR assay.

**Table S5** Primers used in LUC assay.

**Table S6.** Primers used in BiFC assay.

**Table S7.** Primers used in split luciferase complementation assay.

**Table S8.** Primers used in Y2H assay.

**Table S9.** Primers used in pull-down assay.

**Table S1.** Primers used in VIGS

| Vector | | Forward primer (5’-3’) | Reverse primer (5’-3’) |
| --- | --- | --- | --- |
| *DELLA* | gtgagtaaggttaccgaattcCAGATGCTGGAATGGATGAGC | | cgtgagctcggtaccggatccACTTTGTACCCAACCAGCCA |

**Table S2.** Primers used in RT-qPCR

| Gene | Accession number | Forward primer (5’-3’) | Reverse primer (5’-3’) |
| --- | --- | --- | --- |
| *ACTIN2* | Solyc03g078400 | GGTCGGAATGGGACAGAAG | CTCAGTCAGGAGAACAGGGT |
| *PIF1a* | Solyc09g063010 | TCGTGGCTTCATTACCCACT | GACGTACGGTTTTCTCGTGG |
| *PIF1b* | Solyc06g008030 | CATCCAGAATCCCAGTTGCG | CCACTTCCTGATTCTGGGGT |
| *PIF3* | Solyc01g102300 | AGATGCCACATTTCTCCCCAc | TGCACAGAGGGCATTGGATA |
| *PIF4* | Solyc07g043580 | TCCAATGCCACCTCCAAGAT | AAGCTGCACATTGGATGACC |
| *PIF7a* | Solyc03g115540 | ATTCTGTTAGCCCTGCCAGT | TGCTGCCTGTGATCTGTGTA |
| *PIF7b* | Solyc06g069600 | TCCTCCCTTCATGTCACCAC | TGAGTTTGGGGCTGATGGAT |
| *PIF8a* | Solyc01g090790 | TGCTGCTATGCCTGATCCTT | CAGGTACAATGCTGCCATCC |
| *PIF8b* | Solyc01g018510 | GCAGCCCCAACATAACAACA | GGTACAATGCTGCCATCCTG |
| *BCP1* | Solyc10g081520 | GACCGGCTACTAAGGATGATTC | CTCTTGAAGTAGGCAAAGCTCTA |
| *PT4* | Solyc06g051850 | TGATCATACATCAACCAATCTCATC | ACTACAAGATTGTCTGAGGCCA |
| *PT5* | Solyc06g051860 | CCATGTTGTTTTTGGCATTCA | CGGCCAGAGTCAGTCCTATTAC |
| *CCD7* | Solyc01g090660 | AGCAGATTTTCCAGCGATGAA | AAAGGAAAATGGGGTAGCGC |
| *CCD8* | Solyc08g066650 | CCAATTGCCTGTAATAGTTCC | GCCTTCAACGACGAGTTCTC |
| *MAX1* | Solyc08g062950 | GGTGTTACATTGTCTCGCCC | CTCCAAGAGCCAACCAAACC |
| *DELLA* | Solyc11g011260 | TTGTAACGTGGTGGCTTGTG | TGGAAGCTTGTTTGAACGCA |

**Table S3.** Primers used in EMSA assay

| Vector | Forward primer (5’-3’) | | Reverse primer (5’-3’) |
| --- | --- | --- | --- |
| MBP-*SlPIF4* | gatcgagggaaggatttcagaattcATGGGATTTGATCATGAGCTAGTGG | cctgcaggtcgactctagaggatccTCAAGTGGCAGGTGCATTACTATTA | |
| *SlCCD7* | AACTTTAATTTATGACCACATGACAAAAACTCTTT | AAAGAGTTTTTGTCATGTGGTCATAAATTAAAGTT | |
| *SlCCD7*-mut | AACTTTAATTTATGACCGACAAAAACTCTTT | AAAGAGTTTTTGTCGGTCATAAATTAAAGTT | |
| *SlCCD8* | TTTAACGTTAATTTTTCACATGACATACTTAAAATCAC | GTGATTTTAAGTATGTCATGTGAAAAATTAACGTTAAA | |
| *SlCCD8*-mut | TTTAACGTTAATTTTTTTTTTTACATACTTAAAATCAC | GTGATTTTAAGTATGTAAAAAAAAAAATTAACGTTAAA | |
| *SlMAX1* | AGTTTTCGAATGCACGTGTATCCCATGTTA | TAACATGGGATACACGTGCATTCGAAAACT | |
| *SlMAX1*-mut | AGTTTTCGAATGTATCCCATGTTA | TAACATGGGATACATTCGAAAACT | |
| *PT4* | CTACCAGGGTGAGATCCACTTGCCACGCCCTGAGTGCT | AGCACTCAGGGCGTGGCAAGTGGATCTCACCCTGGTAG | |
| *PT4*-mut | CTACCAGGGTGAGATCCGCCACGCCCTGAGTGCT | AGCACTCAGGGCGTGGCGGATCTCACCCTGGTAG | |
| *PT5* | CAAAATGTGTGCAATACACTTGCGGATGACATGTCATG | CATGACATGTCATCCGCAAGTGTATTGCACACATTTTG | |
| *PT5*-mut | CAAAATGTGTGCAATACGCGGATGACATGTCATG | CATGACATGTCATCCGCGTATTGCACACATTTTG | |

**Table S4. Primers used in ChIP-qPCR assay**

| Gene | Forward primer (5’-3’) | Reverse primer (5’-3’) |
| --- | --- | --- |
| *SlCCD7* | AATGCCACAAGATTAAAGGAC | TGGTTTAGTTTGACTTGACACG |
| *SlCCD8* | TTCAAGTCGAGTTAGGTCGTTTCT | TCTTCTTTTTCTTCTTCCTCCGTC |
| *SlMAX1* | GTATAGTTTTCGAATGCACGTG | GCAAGTTGCTATCTTAGTTGAATG |
| *PT4* | GGACATTTGCAGCTCTTTATGG | CACCCCAAATGCACTAACCA |
| *PT5* | ACACTTGCGGATGACATGTC | TGGGGAGGCAAAAGAGAAGT |

**Table S5.** Primers used in LUC assay

| Vector | Forward primer (5’-3’) | Reverse primer (5’-3’) |
| --- | --- | --- |
| SK*-SlPIF4* | GCGGATCCATGGGATTTGATCATGAGCTAGTG | GGGGTACCAGTGGCAGGTGCATTACTATTAGTTG |
| GFP-*SlDELLA* | ctctcgagctttcgcgagctcATGAAGAGAGATCGAGATCGAGATC | gcccttgctcaccatggatccCAACTCGACTTCTCCGGCG |
| LUC-*CCD7* | ggtaccgggccccccctcgagTTAATTAAATTCAGGATTTTAACTATATGTTACC | agaactagtggatcccccgggTACATCACCCTCAACCCCTCC |
| LUC-*CCD8* | ggtaccgggccccccctcgagTGAATATAGCTCACTTATACATAAATTATACTCC | agaactagtggatcccccgggACTCCTATTACATGTAAACAAATGAGGC |
| LUC-*MAX1* | ggtaccgggccccccctcgagTGAAAAGTGGGAATCCTAATCACA | agaactagtggatcccccgggCCAATAAATATAGTGTATTCTACATGATTAAAAG |
| LUC-*PT4* | ggtaccgggccccccctcgagGATTGCCTTGCTCGGTACATTC | agaactagtggatcccccgggTCCATCGTCTTGACGGCCA |
| LUC-*PT5* | ggtaccgggccccccctcgagAATTTTCACTTAGACACCTCAAATAGGT | agaactagtggatcccccgggGCTGAAAATTAGTTGCTTTTTAATAATTT |

**Table S6. Primers used in BiFC assay**

| Vector | Forward primer (5’-3’) | Reverse primer (5’-3’) |
| --- | --- | --- |
| YFP^n^-SlPIF4 | ccttaattaacATGGCAGCAGGGGAGAAGAAAAGCCTGAAT | gactagtGTACTCAAGTGACCCCAGGGGGATCCCATT |
| YFP^C^-SlDELLA | ccttaattaacATGACAAGTATAGTGGGCAATCAGAAAGA | gactagtAACTCCACATCCCAAGCCTCTTGCTTCT |

**Table S7. Primers used in split luciferase complementation assay**

| Vector | Forward primer (5’-3’) | Reverse primer (5’-3’) |
| --- | --- | --- |
| cLUC-SlPIF4 | ggggacaagtttgtacaaaaaagcaggctttATGGGATTTGATCATGAGCTAGTG | ggggacaagtttgtacaaaaaagcaggctttAGTGGCAGGTGCATTACTATTAGTTG |
| nLUC-SlDELLA | ggggacaagtttgtacaaaaaagcaggcATGAAGAGAGATCGAGATCGAGATC | ggggacaagtttgtacaaaaaagcaggctttCAACTCGACTTCTCCGGCG |

**Table S8. Primers used in Y2H assay**

| Vector | Forward primer (5’-3’) | | Reverse primer (5’-3’) |
| --- | --- | --- | --- |
| AD-SlPIF4 | | gccatggaggccagtgaattcATGGGATTTGATCATGAGCTAGTG | cagctcgagctcgatggatccAGTGGCAGGTGCATTACTATTAGTTG |
| BD-SlDELLA | | atggccatggaggccgaattcATGAAGAGAGATCGAGATCGAGATC | ccgctgcaggtcgacggatccCAACTCGACTTCTCCGGCG |

**Table S9. Primers used in pull-down assay**

| Vector | Forward primer (5’-3’) | | Reverse primer (5’-3’) |
| --- | --- | --- | --- |
| MBP-SlPIF4 | | gatcgagggaaggatttcagaattcATGGGATTTGATCATGAGCTAGTGG | cctgcaggtcgactctagaggatccTCAAGTGGCAGGTGCATTACTATTA |
| GST-SlDELLA | | gatctggttccgcgtggatccATGAAGAGAGATCGAGATCGAGATC | gtcacgatgcggccgctcgagCAACTCGACTTCTCCGGCG |
